# Community-dwelling older adults’ perspectives on health risks: a qualitative study exploring anxieties, priorities, and expectations in ageing

**DOI:** 10.1186/s12889-024-18878-z

**Published:** 2024-06-21

**Authors:** Shaoqi Zhai, Zhiying Zhang, Ruyi Zhang, Yingchun Peng, Jiaying Zhang, Yiyao Zhang, Qilin Jin, Jiaojiao Zhou, Jingjing Chen

**Affiliations:** 1 School of Medical Humanities, Capital Medical University, No.10, Xitoutiao, You An Men Wai, Beijing, 100069 China; 2 Outpatient Office, Beijing Luhe Hospital, Capital Medical University, Beijing, 101149 China; 3 Ethics Committee Office, Beijing Ditan Hospital, Capital Medical University, No. 8 Jingshun East Street, Chaoyang District, Beijing, 100015 China; 4 Cardiac Surgery Department, People’s Hospital of Beijing Daxing District, No.26, Huangcun West Street, Daxing District, Beijing, 102600 China; 5 Fengtai District Xiluoyuan Community Health Service Centre, Beijing, 100077 China; 6 Huairou District Liulimiao Community Health Service Centre, Beijing, 101499 China

**Keywords:** Health risk, Health anxiety, Healthy ageing, Integrated care, Qualitative research

## Abstract

**Background:**

With the conflict between the promise of ageing in health and longevity and the limited availability of health resources and social support, older adults in China inevitably experience anxieties surrounding health risks. This study aims to investigate how older adults perceive the health risks that come with getting older, explore the degree to which health risks affect older adults, and advocate for active engagement in practices for managing health risks.

**Methods:**

Using purposive sampling, three districts of Beijing (Xicheng District, Fengtai District, and Daxing District, respectively) were selected for the research. Qualitative semi-structured and in-depth interviews were conducted with 70 community-dwelling older adults who participated in the study. Data were extracted and analyzed based on a thematic framework approach.

**Results:**

Three main themes were identified: (i) the anxieties of older adults concerning health risks in ageing; (ii) the priorities of older adults for health risk management in ageing; (iii) the expectations of older adults for health risk management in ageing. The primary health concerns among older adults included disease incidence and function decline. It was found that basic health management emerged as a critical need for older adults to mitigate health risks. Moreover, it was observed that healthcare support for older adults from familial, institutional, and governmental levels exhibited varying degrees of inadequacy.

**Conclusions:**

The primary source of anxieties among older adults regarding health risks predominantly stems from a perceived sense of health deprivation. It is often compounded by persistent barriers to primary care of priorities in managing health risks among older adults. In addition, the expectations of older adults for health risk management emphasize the necessity for integrated care approaches. Therefore, further research should give priority to the prevention and management of health risks, aim to reduce anxieties, provide integrated care to meet the primary needs and expectations of older adults, and ultimately strive toward the overarching goal of promoting health and longevity.

**Supplementary Information:**

The online version contains supplementary material available at 10.1186/s12889-024-18878-z.

## Background

The World Health Organization (WHO) defines an “aged society” as a demographic wherein individuals aged 65 and above constitute 14 to 20% of the total population, while a “super-aged society” is characterized by this age group representing 20% or more. In 2022, China transitioned into an aged society, with 14.9% of the total population aged 65 and above [1]. As the capital of China, Beijing typifies an aged city while boasting significant characteristics in terms of medical resources and population distribution [2]. The urbanization rate of Beijing reached 87.60% in 2022, with the urbanization process continuing to accelerate. Due to its historical, economic, and social development advantages, urban areas in Beijing have gathered a considerable amount of high-quality medical resources, including leading hospitals and specialized medical teams. These resources serve the urban residents and extend to the surrounding suburbs. The distribution of the ageing population closely correlates with the allocation of medical resources, given that they constitute the primary beneficiaries of such resources.

With the explosive growth of the ageing population, older adults would be affected both physically and psychologically with health risks emerging successively, which can easily evoke their anxieties and fear of unknown health issues. Health anxiety refers to an individual’s abnormal, persistent, and excessive concern about one’s health status, which significantly impacts the quality of life of older adults [3, 4]. Ageing represents the most significant health risk factor. Coupled with the cumulative effects of environmental and genetic exposures to diverse risk factors, older adults are vulnerable to adverse health outcomes, such as disease, functional decline and dysfunction [5]. Failure to monitor and manage health risks among older adults can precipitate poor health outcomes. Previous studies mainly focused on diseases and life expectancy in ageing from the perspective of heredity and biology. According to the *World Health Statistics Report (2021)*, the average life expectancy in China stood at 77.4 years. As global average life expectancy continues to rise, the prevalence of health risks among older adults, such as cognitive, motor, and sensory decline, dramatically surged [6]. The sustained extension of life expectancy underscores the imperative of health risk prevention in ageing as a core issue in public health.

The health risks in ageing affect the quality of life of older adults and impose a heavy burden on families and society. Today, the prevailing trend among most older adults is to age in place, either at home or in a community, making “Ageing in place” the prevailing paradigm for healthy ageing worldwide. The health services (treatment, nursing, and rehabilitation) and other community services (social and welfare) required by older adults are provided primarily within the communities where they reside [7–9]. Older adults may become frail and experience challenges as they age, posing risks to their ability to maintain health and safety at home and in the community. Given the rapid decline in physiological, psychological, and social function, proactive interventions from healthcare systems become imperative to address the needs of older adults at high risk of health issues. It is pivotal to improving their quality of life and ensuring cost-effective care.

Despite promoting Integrated Care for Older People (ICOPE) in numerous countries to cope with the challenges of senior care, older adults are confronted with an awkward situation of longevity but unhealthy lives [7, 10]. A surge in the number of high-risk older patients, coupled with ineffective population health management and the absence of effective preventive measures in the healthcare system to monitor, engenders tremendous cost pressures on society as a whole [10]. Meanwhile, the traditional top-down approach to governance, dominated by the organization, has led policymakers to overlook the ideas and experiences of older adults [11]. However, as direct recipients of services, older adults’ needs and experiences directly reflect the effectiveness and responsiveness of the healthcare system. Unfortunately, policymakers and managers disproportionately focus on structural barriers, resulting in the lack of insights into the demand side in formulating healthcare

policies. Indeed, older adults are concerned about how policymakers respond to their demands for a longer and healthier life [6]. The efficacy of healthcare policies may be compromised due to a lag in responding to the health demands of older adults, underscoring the imperative to explore issues directly from their perspective of the demand side. Consequently, valuable insights from older adults regarding their attitude and management of health risks are indispensable for effectively implementing population ageing health management strategies.

Profound policy reforms related to addressing the challenges of population ageing and improving the quality of person-centred care can help older adults navigate life-cycle risks. How do we consider the impact of health risks on the well-being of the community-dwelling older adults [12]? Before considering policy reform initiatives, policymakers and managers need to capture ideas about older adults concerning ageing and health risks, and what types of services align with their needs [11, 13]. Older adults hold a paramount responsibility for their health, serving as the primary stewards of their well-being. With age comes an enhanced awareness and discernment of health risks, highlighting older adults’ profound insights regarding their own health [14]. Therefore, it is essential to utilize the direct and accurate perceptions of older adults to promote health management strategies aimed at early detection and intervention, ultimately fostering healthier ageing and improving the quality of life [15].

Qualitative methods allow researchers to delve into the multifaceted perspectives of older adults and analyze meaningful associations between health and ageing. Previous qualitative studies have predominantly focused on elucidating family economic vulnerability under the impact of health risks [12, 15, 16], explored the demand and preferences of geriatric care [11, 17, 18], and examined the willingness and obligations of informal caregivers to support older adults [13, 19, 20, 21, 22]. There remains a discernible gap in understanding the impact of health risks in an ageing society from a preventive viewpoint. Closing this gap is imperative for devising comprehensive strategies to mitigate health risks among older adults and foster a conducive environment for healthy ageing.

Due to the progressive development of the global population ageing and the urgent desire of older adults for longevity and health, researchers imperatively need to shift paradigms from “Treatment Of Disease” to “Preventive Treatment Of Disease” [23, 24]. This proactive approach is crucial for tackling the crisis of care dependency caused by health risks. In other countries, the health risks of older adults mainly focus on three aspects: health and function (physical or cognitive decline), lifestyle and behaviour (unhealthy habits or poor self-care ability), and social and physical environment (social isolation, burden or danger of caregivers) [25]. Therefore, integrating and redesigning the quality of person-centred care for older adults from a preventive viewpoint of health risks presents a clear trajectory forward. Taking the communities as research sites, this study was designed to delve into older adults’ perceptions, experiences and wisdom regarding ageing. It seeks to explore older adults’ anxieties, priorities, and expectations concerning health risks and help policymakers better tailor approaches to senior care and improve the quality of older adults. This study is the first to advance geriatric disease prevention and consider a person-centred approach to enhancing care strategies for older adults in China. Furthermore, contributing to the global discourse on this topic offers policymakers in other countries a Chinese paradigm for managing health risks among older adults.

## Methods

### Study design and setting

From December 2021 to January 2022, a qualitative study was conducted with community-dwelling older adults in Beijing, China. Using semi-structured and in-depth interviews, this study explores the perceptions, experiences, knowledge, and values of older adults regarding the health risks in the ageing of their residence. This study selected older adults as the research object because everyone must be responsible for their health. Exploring older adults’ understanding of health risks is beneficial for them in improving their health management level and better coping with the problem of ageing. The one-on-one in-depth interviews facilitate a more personalized and intimate communication between the researcher and participants, provide a flexible space for in-depth study of everyone’s opinions, avoid group pressure and convergence psychology, and improve the accuracy of the results. A qualitative case study method is adopted, and qualitative research follows the Standards for Reporting Qualitative Research (SRQR) to ensure the quality of the article [26].

### Study sample and recruitment

The traffic on the ring road in Beijing centred on Tiananmen Square has played an important role in reflecting the progress of the economy and population ageing among 16 districts. The city has an urban-suburban layout from the inner ring to the 6th ring. We selected three districts based on their ring road location to capture a representative sample: Xicheng District (within the 2nd ring), Fengtai District (3-4th ring), and Daxing District (5-6th ring). Xicheng District is located in the core areas of the old city between the second ring road and Tiananmen, Fengtai District in the sub-central regions between the third and fourth ring roads, and Daxing District in the suburban areas between the fifth and sixth ring roads. We are recruiting participants with purposive sampling by selecting one community from each district as an interview site.

Criteria for inclusion: (i) aged 60 or above, as defined by China’s Law on Older Adults’ Rights and Interests; (ii) living in communities; (iii) being willing and consenting to participate; and (iv) being able to express themselves independently. Exclusion Criteria: (i) could not be independent; (ii) living in nursing facilities; (iii) having mental disorders affecting their expression; or (iv) refusing in-depth interviews.

Our analyses were based on 70 community-dwelling older adults. We recruited 28 men and 42 women aged 60 to 90. Of the 70 interviewees, 55.7% were from the Xicheng District, 21.4% were from the Fengtai District, and 22.9% were from the Daxing District. Approximately 52.9% of the respondents have junior high school education or below, 90% were in multi-person households, and 88.6% were not living alone. Details are shown in Table 1.

**Table 1:** Demographic characteristics of the interviewed older adults

Items	Number of interviewees	Percentage (%)
Gender		
Male	28	40.0
Female	42	60.0
Age		
60∼	32	45.7
70∼	34	48.6
80∼	4	5.7
District		
Xicheng District	39	55.7
Fengtai District	15	21.4
Daxing District	16	22.9
Education		
Elementary school or below	2	2.9
Junior high school	35	50.0
Senior or technical secondary school	18	25.7
Bachelor’s degree or above	15	21.4
Household size		
single-person	7	10.0
2 ∼ 3 persons	37	52.9
4 ∼ 6 persons	24	34.2
7∼ persons	2	2.9
Living Arrangements		
Living alone	8	11.4
Couple living together	27	38.6
Living with their spouse and offspring	26	37.1
Living with offspring (without spouse)	9	12.9
Total	70	100.0

### Data collection

The research group is composed of professors, nurses, and doctors from the healthcare field. Interviews with the community-dwelling older adults were conducted by the research members who have received training in qualitative methods. On each visit to a community, researchers established contact with older adults who came to community health centres and invited those interested as participants. All the participants fully understood the study and signed the informed consent form.

In adherence to ethical guidelines, the interviews were conducted in a secluded meeting room, where participants were duly informed of the utilization of recording devices during the conversations and assured of the confidentiality of their data. The semi-structured interviews were conducted directly, face-to-face, with an average duration of approximately 30 min, following the interview procedures outlined in Supplement File 1. The researchers recorded the audio; field notes were taken to supplement the transcripts. Data saturation was measured from the perspective of both interviewees and researchers. Information saturation was considered achieved when researchers thought that there were no new topics or concepts related to the emerging theme and interviewees expressed themselves ultimately without any relevant supplements [27, 28].

### Data analysis

According to the qualitative data analysis methodology outlined by Nicola K Gale in multi-disciplinary health research, we followed the thematic framework analysis procedure [29]. NVivo (11, QSR) and Excel (Version 16.67) were used to promote data encoding. Eight researchers formed a coding group. First, to ensure the credibility of the data analysis, eight researchers received homogeneous training and underwent two rounds of review after the data analysis was completed. In the first stage, the interview team promptly transcribed the audio recordings of the interviews after the interview. Second, researchers listened to the audio recordings and read the interview notes to familiarize themselves with the knowledge and experiences related to the interviewees’ feelings and behaviours about the topic. Third, every time the interview transcripts reached 10, the researchers meticulously reviewed each one, line by line, and used inductive techniques to code the data set to update the codebook. The cycle was repeated seven times at this stage until the total number of interview transcriptions reached 70, which was already at the point of saturation for all areas combined, and the codebook was repeatedly discussed and revised, with coders reaching a consensus that the codes were stable enough to stop data collection [30]. Fourth, the transcripts were compared, and researchers reached a consensus to group codes into appropriate categories to establish a practical analysis framework. Fifth, the following transcripts were indexed using the available codes and categories, applying the analytical framework. Sixth, representative quotes from the interviews were selected, and their information was inserted into the frame charts. Seventh, the data was analyzed, connections among the data were investigated, and the reasons for the emergence of the phenomenon and its significance were described [31, 32].

## Results

Utilizing thematic framework methodologies, the qualitative research findings have been condensed and refined into three distinct themes, as outlined below:

i anxieties of older adults concerning health risks in ageing;
ii priorities of older adults for health risk management in ageing;
iii expectations of older adults for health risk management in ageing.

### Theme 1: anxieties of older adults concerning health risks in ageing

This study summarized the main health risks perceived by older adults and their attitudes towards these risks, as shown in Table 2.

**Table 2:** The main health risks perceived by older adults and their attitudes towards health risks

Perceived health risks	Specific risk	Example codes of needs	Attitudes to the risks	Example codes of attitudes
Disease (*n* = 30)	Cardio-cerebrovascular risk (*n* = 27)	Sudden death, heart attack, cerebral infarction	Grief and worry	Great pain, regrets, family, suddenly, unacceptable, unprepared, care for family
	Cancer risk (*n* = 3)	Cancer, scared, physical pain, short survival period	Helplessness and fear	Desperation, incurable, fear of death, helpless
Function (*n* = 19)	Disability risk (*n* = 15)	Couldn’t walk, unable to live independently	Disappointment and incompetence	Sense of incompetence, reliance, loss of autonomy, control, and independence
	Dementia risk (*n* = 4)	Dementia, amnesia, forgetting family members and important things	Useless and worthless	Losing myself, meaningless life, bottomless burden, loss of subjectivity and burden on families
None (*n* = 10)		Natural ageing, normal, positive symptom-controlled, compliance	Optimistic	Nothing to worry about death, enjoy the life, focus on things can be changed

#### Subtheme 1: the main health risks perceived by older adults

(i) Diseases are the main identifiable risks perceived by older adults.

Cardiovascular and cerebrovascular diseases are the most frequently mentioned health risk among older adults (*n* = 27). The older adult P47 stated, “*Media often reports cardiovascular and cerebrovascular diseases, and they have a significant impact on our health.*” (75 years old, female, Fengtai District).

Besides, three older adults thought cancer is also a health risk as the older adult P69 said, “*More and more older adults have cancer, which has become one of the main health risk factors.*” (63 years old, female, Xicheng District).

(ii) Functional impairment is a secondary health risk perceived by older adults.

Fifteen older adults mentioned disability risks, such as the older adult P54 said, “*Many older adults are unable to walk, take showers, and live on their own*” (72 years old, female, Xicheng District). Four older adults mentioned dementia risk, such as the older adult P62 said, “*If I get Alzheimer’s, it will be a great burden on my children.*” (74 years old, female, Xicheng District).

(iii) Some older adults do not perceive health risks.

Ten older adults have not identified health risks in their lives, such as the older adult P50 remarked, “*I don’t know health risks in the ageing process, it’s very normal for the health status of elderly people to deteriorate, natural ageing is inevitable.*” (77 years old, male, Fengtai District).

#### Subtheme 2: attitudes of older adults towards different health risks

(i) Feel worried and fear of the disease (*n* = 22).

The older adult P32 admitted, “*I am terrified that if I had sudden death caused by cardiovascular and cerebrovascular diseases, it would be painful for my family.*” (69 years old, male, Xicheng District). The older adult P56 mentioned, “*When it comes to cancer, I will fall into a fear of death and feel helpless.*” (78 years old, male, Xicheng District).

(ii) Feel disappointed and useless (*n* = 16).

The older adult P7 expressed, “*If I cannot eat and walk independently, I will feel disappointed because I have lost my dignity and autonomy*” (61 years old, female, Daxing District). The older adult P67 said, “*If one day I lost my memory and forgot my name and family, I would feel I am a useless person.*”(75 years old, female, Xicheng District).

(iii) Optimistic to health risks (*n* = 8).

The older adult P18 said, “*Take it easy, one is mortal, all we can do is enjoy life and stay away from worries.*” (67 years old, male, Daxing District).

### Theme 2: priorities of older adults for health risk management in ageing

This study summarized the priorities and barriers identified by older adults for health risk management in ageing, as detailed in Table 3.

**Table 3:** Priorities in different management needs by the interviewed older adults

Needs	Goals	Example codes of needs	Barriers	Example codes of barriers
Primary health management (*n* = 32)	Health monitor	Blood pressure and blood glucose monitor, health risk screening, manage in advance	Lack of dynamic health monitoring technology	No health risk screen tools, insufficient integration of electronic health records, cannot generate effective health risk alerts
	Symptom control	Frailty, age-related declines hypertension, hyperglycemia, physical pain	Fragmented healthcare coordination	Complex symptom, variety needs, Lack of information sharing among different healthcare institutions
	Nutrition and function maintenance	Mobility difficulties, hearing impairment, cooking difficulty, chewing difficulty, imbalanced diet	Lack of multidisciplinary teams	Only family doctors, nurses, and public health physicians, insufficient availability of nutritionists and rehabilitation therapists
Medical care management (*n* = 22)	Disease control	Chronic diseases, complications, fear of stomach and oral disease	Limited availability of treatment options	Limited access to certain therapies in community health centres, lack of transparency in information
	Long-term care support	Bedridden, loss in self-care, dementia or disability, burden on family	Lack of caregiver support	None will take care of me, reject to nursing homes, childless, living alone,
Medication management (*n* = 6)	Medication adherence	Wrong medication, taking less, or taking more	Lack of medication knowledge	Forget medication dosing requirements, daily drugs confused easily
	Medication safety	Antihypertensive medications lead to ankle swelling, antidiabetic medications lead to hypoglycemia, medication side effects	Insufficient availability of geriatric medication	Less variety and quantity of drugs in community health centres

#### Subtheme 1: priority needs and goals in health risk management by older adults

(i) Primary health management emerged as the most prioritized need among the interviewed older adults, including health monitoring, symptom control, nutrition, and function maintenance (*n* = 32).

The older adult P69 remarked, “*There is no doubt that diet is a major concern for those receiving home care. We are unable to cook by ourselves due to a lack of ability. Moreover, we are more concerned with our diet, especially those who have diabetes. So it is difficult to maintain a balanced nutrition.*”(63 years old, female, Xicheng District).

(ii) Medical care management was a secondary priority, focusing on disease control and long-term care support (*n* = 22).

The older adult P15 expressed, “*I believe it’s of the highest priority to ensure good medical care. Particularly, I am worried about worsening hypertension leading to stroke. Sometimes, it is easy to cause blockage of blood vessels because of the loss of attention.*” (63 years female, Daxing District).

The older adult P1 expressed, “*Although I can still take care of myself now, I know that one day I will become disabled and will certainly be eager to get a helping hand from someone. However, I am afraid of the absence of care in the future, and I don’t want to go to a nursing home.*” (63 years old, female, Daxing District).

(iii) Medication management turned into the third priority, with older adults highlighting challenges related to medication adherence and safety (*n* = 6).

The older adult P43 explained, “*There are too many types of medications to take every day, which makes it easy to make mistakes. Potential risks involved in taking the wrong dosage or missing doses altogether.*” (64 years old, male, Fengtai District).

#### Subtheme 2: barriers to prioritizing health risk management by older adults

(i) The lack of dynamic health monitoring technology, fragmented healthcare coordination, and insufficient multidisciplinary teams limit older adults’ access to primary health management.

The older adult P13 stated, “*It is so outdated for the current technology on assessing geriatric health risks that I am unaware of changes in my health status. I believe that individuals do not need to be informed of their ratings, but they want to be told what to pay attention to and what improvements should be made after risk assessment.*” (77 years old, male, Daxing District).

The older adult P53 mentioned, “*I am pretty attentive to screening for diseases, but I often encounter situations where I have to undergo new tests at a different hospital because medical records are not shared between different hospitals.*” (74 years old, male, Fengtai District).

Additionally, the older adult P12 said, “*There are no specialized nutritionists or nutrition clinics at the community health service centre. We mainly have to consult with family doctors for nutritional advice.*” (74 years old, male, Daxing District).

(ii) The limited availability of treatment options and lack of caregiver support hinder medical care management.

The older adult P56 stated, “*If you fall ill, there’s no one to look after you, and hospitalization is beyond affordability.”* (78 years old, male, Xicheng District).

(iii) The barriers to medication management for older adults include a lack of medication knowledge and insufficient availability of geriatric medication.

The older adult P63 said, “*There are numerous medications to take daily, and sometimes it’s hard to track them all. Moreover, the medications related to geriatric conditions are less and available in smaller quantities at the community health service centre.*” (81 years female, Xicheng District).

### Theme 3: expectations of older adults for health risks management in ageing

By using a thematic framework approach, this study synthesized the aspirations of older adults about the services related to controlling health risks. The subjects of service providers were grouped into family, institution and government, as shown in Table 4. Regarding the different types of services of each subject, we coded and listed the details of service provision for each category.

**Table 4:** Providers and offerings expected by older adults for health risks management

Providers	Offerings	Example codes offerings
Family	Day-care	Daily meals, shopping, bath, household activities
	Spirituality	Companionship, chat, greetings, filial piety
	Expenditure	Monthly maintenance, daily living products, medical and care costs
	Non-dependent on children	Self-care, avoid burdening the children
Institution	Healthcare	Clinic visits, medication supervision more drug supply, more on-site care, easy access to rehabilitation therapy
	Service	Lower nursing home admission charges, high-quality caregiver services, available hospice services, more day-care services
	Activity	Diet and activity guidance, more entertainment programs, more health talks
Government	Policy	Free health check-ups, convenient medical services, easy access to long-term care services and home care services, more traditional Chinese medicine services
	Finance	Pensions, increase in the amount of medical insurance reimbursement for critical illness, Lower medical fees, disability allowance
	Facility	Low-cost nursing homes, community ageing stations, ageing-friendly facilities

#### Subtheme 1: family support for older adults in health risks management

(i) The expectation of older adults is mainly rooted in the younger generation, but the reality is that children play a lesser role in caring for older adults.

The older adult P56 said, “*The children should care more about older adults to prevent health risks. But the children often come back late from work and have no extra attention to managing health risks in ageing.*” (78 years old, male, Xicheng District).

(ii) Spouses are the primary caregivers of older adults in managing health risks, but older adults tend to care independently.

The older adult P33 mentioned, “*My spouse helps me more in daily life because my children are occupied. It is good that I can take control of my health by myself.*” (74 years old, male, Xicheng District).

(iii) Older adult couples provide care to each other to prevent health risks but have limited caregiving capacity due to both getting older.

The older adult P65 thought, “*I didn’t live with my children, so I had to support each other with my spouse. But we are both older adults, and it is more difficult to consider each other physically.*” (70 years old, male, Xicheng District).

#### Subtheme 2: institution support for older adults in health risks management

(i) Family doctors play a vital role in controlling the health risks caused by ageing.

The older adult P24 said, “*The community health centres should provide more on-site care for the older adults. For instance, family doctors can provide laboratory services at home for the disabled older adults.*” (60 years old, female, Xicheng District).

(ii) The older adults desire lower nursing home admission costs, high-quality care, and accessible hospice services for managing health risks in ageing.

The older adult P22 mentioned, “*The nursing homes should lower fees, be built as close as possible to the residential community and be equipped with professional medical staff.*” (76 years old, male, Xicheng District).

(iii) The neighbourhood committee should give more attention to organizing community activities such as entertainment programs and health talks for community-dwelling older adults.

The older adult P3 hoped, “*The neighbourhood councils should be proactive in promoting entertainment and health activities for us.*” (67 years old, female, Daxing District).

#### Subtheme 3: government role in health risks management for older adults

(i) Health policies required updating to achieve a more harmonious balance between the escalating health risks and the growing trend of population ageing.

The older adult P1 recommended, “*The government should promote the community-home-care services system in urban and rural areas to offer well-rounded service for the older adults.*” (63 years old, female, Daxing District).

(ii) Increasing the coverage of disability subsidies is an urgent desire of older adults in different regions.

The older adult P22 believed, “*There is a disabled older adult in a family, the economic and care pressure of the whole family will be extremely high, and the government should give some allowance.*” (76 years old, male, Xicheng District).

(iii) Equipping older adult-friendly facilities is an effective approach to address the lack of existing care resources.

The older adult P64 admitted, “*It is urgent to build more nursing homes, due to no child and high cost for the community-dwelling older adults.*” (77 years old, male, Xicheng District).

## Discussion

This study is one of the rare qualitative studies that focus on the prospective impact of health risks in the progression of ageing. It contributes to an extended understanding of the relevant field of research, especially with regard to proactive preventive approaches to health risk management, as seen from the perspective of older adults. Employing a bottom-up approach to capture the internalized values and experiences, this study explored community-dwelling older adults’ anxieties, priorities, and expectations of health risks in ageing, which facilitated the alignment of service delivery with the genuine needs of older adults. Furthermore, the findings underscore the paramount anxieties and expectations of older adults regarding the crisis of care dependency, offering valuable evidence for policymakers to design integrated care and enhance support at the familial, institutional, and governmental levels.

### Health deprivation serves as the primary source of health risk anxieties among older adults

The source of anxiety about health risks among older adults is mainly a sense of health deprivation. Health risks can occur in multiple domains of older adults’ lives, implying uncertainty. As they age, older adults experience a sense of health deprivation, such as physical or cognitive decline and social isolation, which may lead to anxieties. The results showed that cardiovascular diseases were the most worrying health risks perceived by older adults, with 27 interviewees expressing their anxieties about cardiovascular diseases due to the rapid, severe, and poor prognosis. Similar findings can be found in epidemiological studies. Age acts as an individual risk factor for cardiovascular disease, which remains the predominant cause of disability or death among older adults. And now it ranks first globally regarding mortality and morbidity [33]. In addition, inevitable risks of functional decline due to disease or ageing contribute more prominently to the impact of health for older adults, particularly referring to the caregiving dilemmas associated with the onset of disability and dementia [34]. Over 200 million older adults live in China, but 4 million have a disability [35]. Consistent with previous research, disabled older adults could aggravate the burden on family caregiving, especially in finance [36]. Meanwhile, anxieties about health risks among older adults were commonly related to concerns for pain and burden to family members and powerlessness about the loss of self-care. In two studies based on a healthy cohort of older adults in rural Shandong Province, China, findings suggest that substantial social loss (e.g., widowhood or divorce, child loss) amplifies health risks associated with ageing. It is an effective method to help reduce the risk of psychological distress in community-dwelling older adults by screening for frailty and multimorbidity [37, 38]. When exploring anxieties about health risks in older adults, death anxiety is undoubtedly a significant and distinct type. It serves as a psychological response to older adults’ health risks and a complex emotion linked to their perspectives on life, values, and life experiences. It is interesting to note that a significant proportion of older adults have discussed the issue of life and death in this study. Ten older adults mentioned they didn’t worry about health risks, contrary to the findings of previous studies [39, 40]. In the past, discussing death was typically treated as a cultural taboo in China; older adults tended to avoid talking about it [40]. Death of health risks has long been an inescapable fear for older adults in China [39]. However, some interviewees in this study argued that older adults have reached the final stage of life, believing that death is a natural part of life. A study of the attitudes toward death in Chongqing, China, reported a predominantly neutral acceptance of death among older adults [41]. It indicates that some older adults hold optimistic attitudes toward death and are not trapped in health anxieties. Another study from the UK found that death anxiety levels among older adults decreased with age [42]. Some interviewed older adults held positive attitudes toward their health risks and good adherence to self-health management. They believed that the key to a long and healthy life is the practice of autonomy, so self-care enables older adults to enjoy better the changes and meaning of life [43, 44].

### Expanding access to the priorities in health risk management of ageing

The health risk management for older adults presents a multifaceted challenge, encompassing diverse priorities and enduring obstacles. A pivotal understanding of their key needs and barriers is imperative for devising impactful strategies to mitigate the progression of these risks. Primary health management stands out as the foremost priority in health risk management for older adults. This encompasses various elements, including health monitoring, symptom management, and maintenance of nutrition and functional capabilities. Older adults notably express concerns about their ability to monitor vital health parameters such as blood pressure and blood glucose levels, alongside the necessity for timely health risk screenings [45]. Additionally, older adults stress the significance of addressing symptoms associated with frailty, age-related declines, hypertension, hyperglycemia, and physical pain. Furthermore, maintaining adequate nutrition and functional abilities is critical to primary health management. It is evident that older adults prioritize interventions to promote overall well-being and enhance their capacity for life independence and quality of life. Despite recognizing these priorities, numerous barriers impede older adults’ access to effective health risk management [46]. One significant barrier is the lack of dynamic health monitoring technology, which hampers timely assessment and management of health risks [47]. Fragmented healthcare coordination exacerbates this issue, leading to disjointed care delivery and suboptimal outcomes. The absence of multidisciplinary teams further compounds the problem, limiting access to specialized care and tailored interventions. Moreover, the limited availability of treatment options, particularly in community health centres, undermines the effective control of chronic diseases. Additionally, the lack of caregiver support poses challenges in ensuring adequate long-term care for older adults, contributing to feelings of vulnerability and social isolation [48].

### The expectations of older adults for health risk management emphasize the necessity for integrated care

Addressing the priorities and barriers identified in elderly health risk management requires a multifaceted approach involving various stakeholders, including family caregivers, institution providers and government policymakers. This study found that older adults’ informal support networks are primarily made up of their spouses and themselves, while the care from their children is absent. Family is the foundation of health risk management, and adequate family support plays a major part in preventing health risks for older adults. Currently, older adults expressed their anxieties deprived from the loss of self-care, being bedridden, and being unattended due to limited family support. Dementia was specifically mentioned by the older adults interviewed, with the older adult P63 stating, “*I think that getting dementia is the scariest thing; loss of self-awareness and executive function is meaningless for me and bottomless for my family.*” In China, older adults with dementia primarily rely on home care, which involves various health risks. However, the fact is that available services are missing to support dementia older adults and their families in the community [41, 42]. Even if they are caught in financial difficulties or fail to self-care by themselves, they avoid seeking help from their children [49]. At the same time, institutions are crucial platforms in managing health risks in older adults, but they should improve person-centred health management services. In terms of health management services, there are differences between supply and demand perspectives. From the view of the service providers, “Care management” in primary care is adequate. From the perspective of older adults mentioned in interviews, what they want is “caregiving management,” but not the same as “care management.” Unlike “care management,” which is specialized management by medical practitioners, the “caregiving management” that older adults expect is more integrated and continuous, covering all the caregiving needed in their daily lives. It aligns with the idea of Integrated Care for Older People (ICOPE) presented in previous studies, which helps older adults pay attention to their health risks and diverse care needs such as living and recreation [50]. Moreover, the government is like an umbrella in health risk management for older adults, while the implementation of relevant policies still encounters some challenges. Nowadays, the integrated medical and care model has been implemented in China, but the accessibility of integrated care is restricted in communities, mainly remaining at the institutional level [10]. Consequently, the expectation of older adults for health risk management is integrated care, which needs joint efforts from family, institutions, and government to prevent health risks of older adults and improve their quality of life.

### Policy implications

Policymakers are supposed to strengthen the interconnections between families, institutions, and government and establish dedicated centres to bring support to health risk services for older adults effectively. In particular, it is vital to draw more attention to ageing vulnerable groups and establish a multi-subject cooperation mechanism to promote communication collaboration and resource exchange among different subjects [36]. The Sharing Care model of multi-subject collaboration is seen as a solution to the trade-off between caregiving, work, leisure, and paying for care, sharing the burden of care in balancing respite care, caring, and paying for care [13]. Although there may not be a nursing home near every community in Beijing, community health centres and neighbourhood councils have been built. The community is still the central node for providing and coordinating health risk services for older adults in China. In consideration of China’s healthcare system in the future, it’s essential to enrich the medical and care resources in the community and strive to improve the capacity of the community to offer integrated services for older adults. Besides, encouraging older adults to take advantage of community resources for health management will help them and their families better cope with the impact of health risks in ageing [51].

### Limitations

Although this study addresses some key questions about health risks in ageing, there are some limitations. On the one hand, the representativeness of our study was limited since only a sample of community-dwelling older adults in three districts chosen from seventeen in Beijing were interviewed, and participants were from urban and suburban areas. This study didn’t choose rural areas as research sites because the supporting senior care resources in urban areas are more comprehensive, which is more conducive to identifying and responding to health risks. On the other hand, this study conducts relevant research only in Beijing, but we believe it is worth considering more similar studies in smaller cities across China in the future.

### Strengths

Taking the community as a research site, this study selects community-dwelling older adults for personal in-depth interviews, focuses on the impact of health risks in the progression of ageing, investigates older adults’ anxieties, priorities, and expectations about health risks, and then helps policymakers better tailor approaches to integrated care and improve the quality of older adults’ lives. This study, on the other hand, is the first to move geriatric disease prevention forward and consider a person-centred approach to improving older adults’ care strategies in China. It can garner greater attention for pertinent global issues and provide policymakers in other countries with a Chinese paradigm about managing health risks for older adults.

## Conclusions

Currently, Chinese older adults face a dilemma of longevity but unhealthy lives. Findings from this study show that the source of anxieties of older adults concerning health risks in ageing mainly is a sense of health deprivation. Besides, this issue is frequently exacerbated by persistent obstacles in accessing primary care priorities for the management of health risks among older adults. The expectation of older adults for health risk management is integrated care. Therefore, further research should concentrate more on the prevention and management of health risks, eliminate anxieties, provide integrated care to meet the primary needs and expectations of older adults, and finally achieve the goal of health and longevity of older adults.

### Electronic supplementary material

Below is the link to the electronic supplementary material.

Supplementary Material 1

## Data Availability

The datasets generated and analysed during this qualitative study are not publicly available. This is to protect the privacy and confidentiality of participants. However, they are available from the corresponding author upon reasonable request.
