# Supplementary material for: Community-dwelling older adults’ perspectives on health risks: a qualitative study exploring anxieties, priorities, and expectations in ageing
Source: BMC Public Health. 2024 Jun 21;24:1657. doi: 10.1186/s12889-024-18878-z (PMC11191137; doi:10.1186/s12889-024-18878-z)
Supplement: Supplementary file 1 — Supplementary Material 1 [file 12889_2024_18878_MOESM1_ESM.docx]

**Supplement file.**

**Interview questions and**[**prompts**](https://www.sciencedirect.com/topics/nursing-and-health-professions/tylenol)

| Procedures | Outline |
| --- | --- |
| Opening question | Most older adults face the dilemma of unhealthy longevity, and the accelerated ageing process continues to increase the health risks of the ageing population. Please describe your experience, perspectives, or more about the issues of health risks in ageing. |
| Noticing and interpreting | 1. What diseases are you suffering from?  2. What perceptions do you hold of the health risks in ageing  3. Do you have concerns about the possible health risks that may emerge in the future?  4. Can you tell me what health risks you have the most concerns about? What caused you to this concern?  5. What is your top priority for health risk management in ageing? Why?  6. What are your expectations when managing health risks in ageing? In which fields? What are the specific needs? |
| Prompts | What else? Tell me more…. Can you explain the reasons for this? What are your concerns? What is your value? What is your experience? |
